# Genetic Aberrations in Normal Tissues Adjacent to Biliary Tract Cancers

**DOI:** 10.3390/biomedicines13112812

**Published:** 2025-11-18

**Authors:** Chae Hwa Kwon, Sung Hee Park, Hye Ji Lee, Jong Hyun Lee, Sung Yong Han, Yong Mok Park, Hyung Il Seo, Dong Uk Kim, Byeong Gwan Noh

**Affiliations:** 1 Biomedical Research Institute, Pusan National University Hospital, 49241 Busan, Republic of Korea; 2 Department of Internal Medicine, Pusan National University College of Medicine, 50612 Yangsan, Republic of Korea; 3 Department of Surgery, Pusan National University College of Medicine, 50612 Yangsan, Republic of Korea; 4 Department of Internal Medicine, Gumi Medical Center, CHA University, 39233 Gumi, Republic of Korea

**Keywords:** biliary tract cancers, normal tissues adjacent to tumors, field cancerization, genomic alteration

## Abstract

**Background**: The role of normal tissues adjacent to tumors (NATs) in biliary tract cancer (BTC) remains unclear, despite their potential contributions to field cancerization. **Methods**: Targeted genomic profiling of tumor tissues, patient-matched NATs, and peripheral blood leukocytes from 13 patients with BTCs was performed. Clinicopathological data, including inflammatory conditions and precursor lesions (biliary intraepithelial neoplasia [BilIN] and intraductal papillary neoplasm of the bile duct), were integrated with genomic findings. **Results**: Tumor tissues exhibited recurrent alterations in genes regulating DNA damage response, cell cycle control, and oncogenic signaling. Importantly, rather than being genetically silent, NATs harbor early somatic variants distinct from those in both tumor and germline DNA. These alterations were not directly associated with cancer-related pathways, but rather with extracellular matrix-receptor interactions, suggesting that NATs may represent an intermediate step in carcinogenesis. All patients with extrahepatic cholangiocarcinoma presented with BilIN in adjacent tissues, providing histological evidence of field cancerization linked to chronic inflammation. **Conclusions**: This systematic comparison of tumors, NATs, and germline DNAs in BTCs revealed that NATs contain biologically relevant somatic mutations. The concordance between the inflammatory background, precursor lesions, and genomic alterations supports a multistep carcinogenic model and highlights opportunities for early BTC detection and risk stratification.

## 1. Introduction

Genomic alterations are fundamental causes of the initiation and progression of human cancers [1]. Chronic inflammation is a critical mediator of carcinogenesis in biliary tract cancers (BTCs) and creates a microenvironment that promotes malignant transformation [2,3,4]. This persistent inflammatory condition provides the biological basis for the concept of “field cancerization,” a phenomenon where histologically normal-appearing tissues accumulate genetic alterations that predispose them to neoplastic change [5,6,7,8]. Recent large-scale sequencing studies have begun to uncover pre-neoplastic genomic and epigenetic alterations in histologically normal bile duct epithelium, suggesting that chronic inflammation and DNA-repair defects may create a permissive field for malignant transformation [9,10,11,12].

Although normal tissues adjacent to tumors (NATs) are often regarded as genetically intact, the lines of evidence from several studies have indicated that they may harbor early genomic abnormalities [13,14,15]. However, the precise genetic status and biological significance of NATs in BTCs remain largely unknown. In particular, whether NATs contain distinct alterations compared to tumor tissue and germline DNA and whether such changes contribute to the earliest steps of carcinogenesis remain unknown.

Therefore, we performed a comprehensive genomic analysis of paired tumor tissues, NATs, and peripheral blood leukocytes from patients with BTCs. This approach enabled us to identify shared and distinct genetic events in paired tissues, providing insights into the molecular basis of field cancerization and its potential role in early carcinogenic processes in BTCs.

## 2. Methods

### 2.1. Patients and Samples

The samples included surgically resected tumor tissues and paired NATs, as well as peripheral blood from 13 patients diagnosed with BTCs from 2018 to 2019. Of these patients, four had distal cholangiocarcinomas (dCCAs), two had perihilar cholangiocarcinomas, three had intrahepatic cholangiocarcinomas (iCCAs), and four had gallbladder cancer. Adjacent normal tissues were procured at least 5 mm away from the tumor boundary, and hematoxylin-eosin slides were reviewed by two pathologists to confirm the absence of dysplasia or carcinoma. All biospecimens used in this study were obtained from the Pusan National University Hospital Biobank in accordance with the applicable ethical and legal requirements. The use of human-derived materials was approved by the Institutional Review Board of the Pusan National University Hospital (approval number: H-H-1801-020-062). All samples were de-identified before distribution, and no personal identifiers were accessible to the research team. Written informed consent was obtained from all donors by the Pusan National University Hospital Biobank at the time of sample collection in compliance with the Declaration of Helsinki and relevant national regulations.

### 2.2. Sequencing

The 13 paired tissues and blood samples were analyzed using next-generation sequencing. All tissue samples were frozen in liquid nitrogen within 30 min of surgical resection and stored at −80 °C until DNA extraction. DNA was purified using a QIAamp DNA Mini Kit (Qiagen, Hilden, Germany). Tumor tissues were meticulously procured and subjected to whole-exome sequencing (WES), while NATs were analyzed using whole-genome sequencing (WGS). Peripheral blood specimens were also subjected to WES for use as a reference. All samples were carefully matched and paired to ensure robust pairing integrity for subsequent comparative analyses.

### 2.3. Bioinformatic Data Processing and Analysis

The raw sequencing reads were aligned to the human reference genome (GRCh38) using the Burrows–Wheeler Alignment tool (version 0.7.15) with the “-M” option to maximize mapping specificity and precision [12]. After alignment, the resulting binary alignment map (BAM) files were rigorously sorted and indexed using SAM tools (version 1.9). Duplicate reads, which could introduce artifacts into downstream analyses, were stringently identified and removed using Picard (version 2.9.0) in the MarkDuplicates module (http://broadinstitute.github.io/picard/ accessed on 20 October 2023). Due to the critical role of read quality in variant detection, mapping quality recalibration was conducted using the BaseRecalibrator and ApplyBQSR tools from the Genome Analysis Toolkit (GATK).

### 2.4. Identification of Somatic Single-Nucleotide Variants and Short Indels

The Mutect2 caller in GATK (version 4.1.0.0) was used to identify somatic single-nucleotide variants (SNVs) and short insertions/deletions (indels) in tumor tissue and NAT samples. WGS data from the NAT samples were tailored to mirror the WES data by specifically targeting the exonic region using SAM tools to ensure analytical uniformity across both sequencing platforms. WES data from normal blood samples were used as matched controls for comparative analysis.

During post-call filtering, stringent criteria were imposed to ascertain the accuracy of identified variants. In the variant call format file, only mutations marked as “PASS” in the FILTER column with a tumor read count (from the BAM file) exceeding 5 were retained. To identify clinically significant alterations, variants were further refined based on their prevalence in prominent population databases. Mutations with an allele frequency below 0.001 in the 1000 Genomes Project, gnomAD, and Korea1K databases were considered rare variants and selected for further study.

All variants annotated as “pathogenic” or “likely pathogenic” were confirmed to be somatic, as they were absent in matched germline (blood) DNA. The ClinVar pathogenicity classification was retained for functional interpretation, while COSMIC nomenclature (“driver/oncogenic”) was additionally applied to indicate somatic context.

In addition, to ensure the accuracy of *MUC16* variant calls—given the high sequence homology within the *MUC* gene family—raw sequencing reads were manually inspected using the Integrative Genomics Viewer (IGV). This manual curation confirmed unique alignment of the variants to the *MUC16* locus (chromosome 19q13.42) without evidence of cross-mapping to other *MUC* family members.

### 2.5. Calculation of the Tumor Mutation Burden

The tumor mutation burden (TMB) quantifies the total number of somatic mutations per megabase in the tumor-coding region. In this study, we used previously identified somatic SNVs and indels to compute the TMB of the tissue samples. The raw mutation count was normalized by dividing the value by the total length of the exon region, resulting in a TMB metric that reflects the number of mutations per megabase.

### 2.6. Mutational Signature Analysis

Mutational signature analysis was performed to identify the mutational processes that influenced the samples. By considering the premise that specific mutagenic events imprint “signatures” on the genome, insights into the historic mutagenic exposures of a tumor can be gained. The somatic SNVs and indels identified in the tumor samples were used as input data in the deconstructSigs tool to generate a mutational signature. By juxtaposing our findings with established mutational signatures in the COSMIC database, we determined the contribution of the recognized mutagenic processes in our samples.

### 2.7. Copy Number Variation Analysis

Two distinct sample–control pairs were used to detect and analyze copy number variations (CNVs). The first pair involved a comparison of the WGS data of NAT samples with the Korea1K WGS dataset, which served as the normal control. The second pair included a tumor sample and a matched normal blood control, both of which were subjected to WES.

The bioinformatics pipeline for CNV detection was anchored using the CNVkit.py tool, which was designed to identify genomic alterations from sequencing data. Following the initial CNV detection, a statistical filter was applied to refine the results, retaining only the CNVs that achieved a *p*-value of less than 0.05 in a two-tailed t-test, thereby emphasizing the most statistically significant differences between the tumor and normal samples.

In addition to broad CNV analysis, a focused examination of focal CNVs was conducted using the GISTIC2 algorithm. This allowed for the identification of genomic regions with significant gains or losses that may play a critical role in tumorigenesis or other disease processes. GISTIC2 analysis is instrumental in identifying recurrent CNVs that may have biological and clinical importance.

### 2.8. Pathway and Network Analysis

Kyoto Encyclopedia of Genes and Genomes (KEGG) pathway enrichment was performed using the DAVID platform (v2024.2), and pathways with *p* < 0.05 were considered significant. To further assess biological connectivity among the mutated genes, an interactome network was constructed using the STRING database (v12.0). The network included 193 unique protein-coding genes after removal of redundant identifiers from the KEGG output. Interaction confidence thresholds were set at > 0.6 (high confidence), and experimental evidence filters were applied to distinguish validated from predicted interactions. Network topology parameters, including the number of edges, average node degree, and clustering coefficient, were analyzed using Cytoscape (v3.10).

## 3. Results

### 3.1. Patient Characteristics

Among the 13 patients, four presented with inflammatory or predisposing conditions, such as gallstones, hepatolithiasis, gallbladder polyps, and viral hepatitis. Histopathological evaluation confirmed that all tumors were adenocarcinomas, predominantly of moderate differentiation. Importantly, associated precursor lesions were detected in all patients with extrahepatic cholangiocarcinoma (EHCCA), including biliary intraepithelial neoplasias (BilIN) 1–3 and intraductal papillary neoplasms of the bile duct with high-grade dysplasia, highlighting the contribution of multistep carcinogenic processes. Resection margins were negative (R0) in 11 patients, with two showing microscopically positive margins (R1) (Table 1).

### 3.2. Somatic Mutations

**Figure 1:**
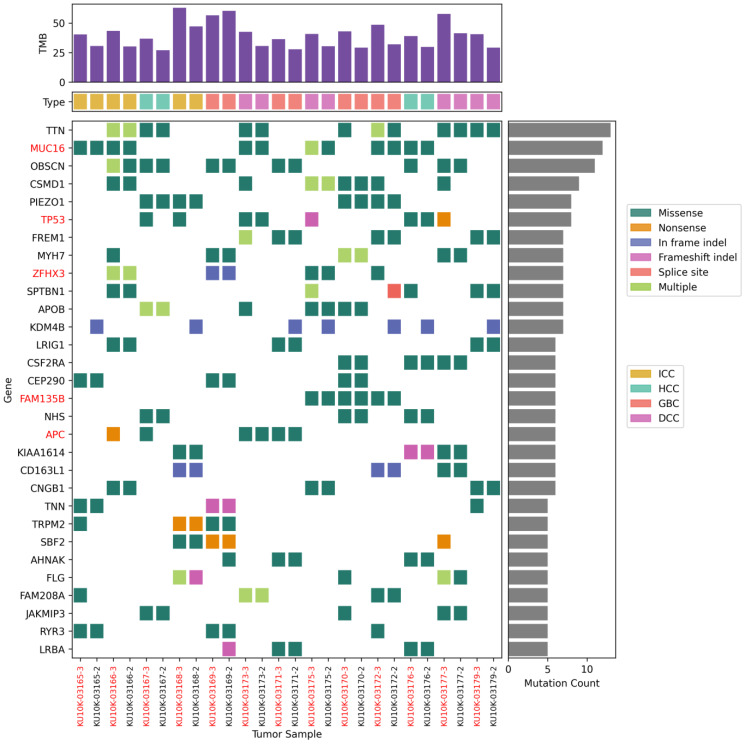
Top 30 most frequently mutated genes. Somatic mutations of single-nucleotide variants and indels in patients with biliary tract cancers.

The somatic mutations were profiled in 13 paired tissue samples (Figure 1). The number of mutations in NAT samples was lower than that in the corresponding tumor tissues. We identified an average of 175 SNPs (range 121–290) and 16 indels (range 10–25) in NATs and 247 SNPs (range 161–437) and 23 indels (range 9–31) in tumor tissues. Tumor tissues exhibited a significantly higher number of mutations compared with NATs (*p* = 0.021). However, no differences were found in the number of mutations or mutated genes according to the BTC tumor type. The most frequently mutated gene was titin (*TTN*; 13/26, 50%), followed by other mutated genes occurring at over 30% frequency, such as mucin 16 (*MUC16*; 12/26, 46%), obscurin (*OBSCN*; 11/26, 42%), CUB and Suchi multiple domains 1 (*CSMD1*; 9/26, 35%), and Piezo type mechanosensitive ion channel component 1 (*PIEZO1*; 8/26, 31%). Most of these frequently mutated genes are unknown cancer drivers. These mutations are commonly observed in tumor tissues and their corresponding NATs.

Tissue-specific mutations were also identified. An in-frame insertion mutation (c.2901 + 62_2901 + 63 ins) in lysine-specific demethylase 4 B (*KDM4B*; 7/26, 27%) was observed in seven patients with NATs. In contrast, *TP53* mutations (8/26, 30%) were predominantly observed in tumor tissues. Mutations p.Q136E and p.P151A in *TP53* were observed in both NATs and tumor tissues from patients S424 and S427, respectively. Different *TP53* mutations were found only in the tumor tissues of four other patients: p.G262V in patient S390, p.P278T in patient S430, p.C229Yfs*10 in patient S426, and p.E258* in patient S028.

Next, cancer-causing genes listed in the Cancer Gene Census (CGC) of the COSMIC database were analyzed. In total, 33 mutations in 13 genes were shared by NATs and tumor tissues from two or more patients (Table 2). The most frequent mutations were those in *TP53* in 6 of the 13 patients (46%). Interestingly, paired tissues from patient S424 shared 13 mutations in 12 CGC genes, including p.Q136E in *TP53*, p.K939T in adenomatous polyposis coli (*APC*), p.Y276C and p.S573E in SMAD family member 4 (*SMAD4*), and p.G1470S in polybromo 1 (*PBRM1*). Paired tissues from patients S429 and S431 shared p.L2038V in *APC* and p.Y352* in E74-like factor 3 (*ELF3*), respectively. Well-known pathogenic mutations were only detected in the tumor tissues (Table 3): p.G12D in *KRAS* in patients S428 and S430, and p.E545K in phosphatidylinositol-4,5-bisphosphate 3-kinase catalytic subunit alpha (*PIK3CA*) in patient S170. Indeed, patients S428 and S170 exhibited likely pathogenic mutations, namely p.S1539* in *APC* and p.X268_splice in phosphatase and tensin homolog (*PTEN*), respectively. Patient S431 also harbored likely pathogenic mutations such as p.D108N in cyclin-dependent kinase inhibitor 2A *CDKN2A* and p.S310F in erb-b2 receptor tyrosine kinase 2 (*ERBB2*). Unlike other pathogenic mutations, p.P151A in *TP53* was identified in both NAT and tumor tissues from patient S427.

### 3.3. Pathways Associated with the Mutant Genes

**Figure 2:**
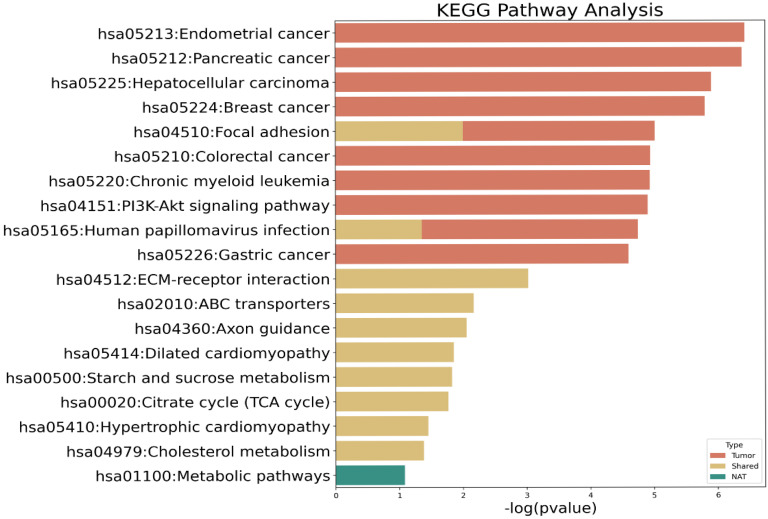
Kyoto Encyclopedia of Genes and Genomes (KEGG) pathway analysis of mutated genes. Red bars, mutated genes in tumor tissue; yellow bars, shared mutations between normal tissue adjacent to tumors (NAT) and tumor tissue; green bar, mutated genes in NAT.

Next, we conducted KEGG pathway analysis to determine whether the mutant genes identified in only NATs or tumor tissues, or those shared by the paired tissues, were related to a specific biological mechanism (Figure 2 and Supplementary Table S1). Mutant genes in NATs were associated with metabolic pathways, albeit not significantly. Mutations shared by NATs and tumor tissues were significantly associated with extracellular matrix–receptor interactions and focal adhesion pathways. Moreover, shared mutations were enriched in citrate cycle pathway-related genes such as isocitrate dehydrogenase (NADP+) 2 (IDH2) and isocitrate dehydrogenase (NAD+) 3 non-catalytic subunit beta (IDH3B). Mutant genes in tumor tissues are significantly associated with various cancer types, such as endometrial, pancreatic, hepatocellular, breast, colorectal, and gastric cancers, as well as the phosphatidylinositol 3-kinase–protein kinase B (PI3K–Akt) signaling pathway, which is a major signaling pathway in cancer.

To complement the KEGG analysis, we examined the protein–protein interaction (PPI) network of 193 residual genes using STRING (confidence > 0.6). The interactome contained 4946 total edges, of which only 99 (≈2%) were supported by experimental evidence, while the remainder represented computational or text-mining predictions. This finding indicates that the majority of inferred connections remain hypothetical and should be interpreted with caution. Nonetheless, experimentally validated interactions clustered around key hubs such as TP53, PIK3CA, and SMAD4, which are canonical regulators of DNA-damage response and oncogenic signaling in BTCs.

### 3.4. Mutational Signatures

**Figure 3:**
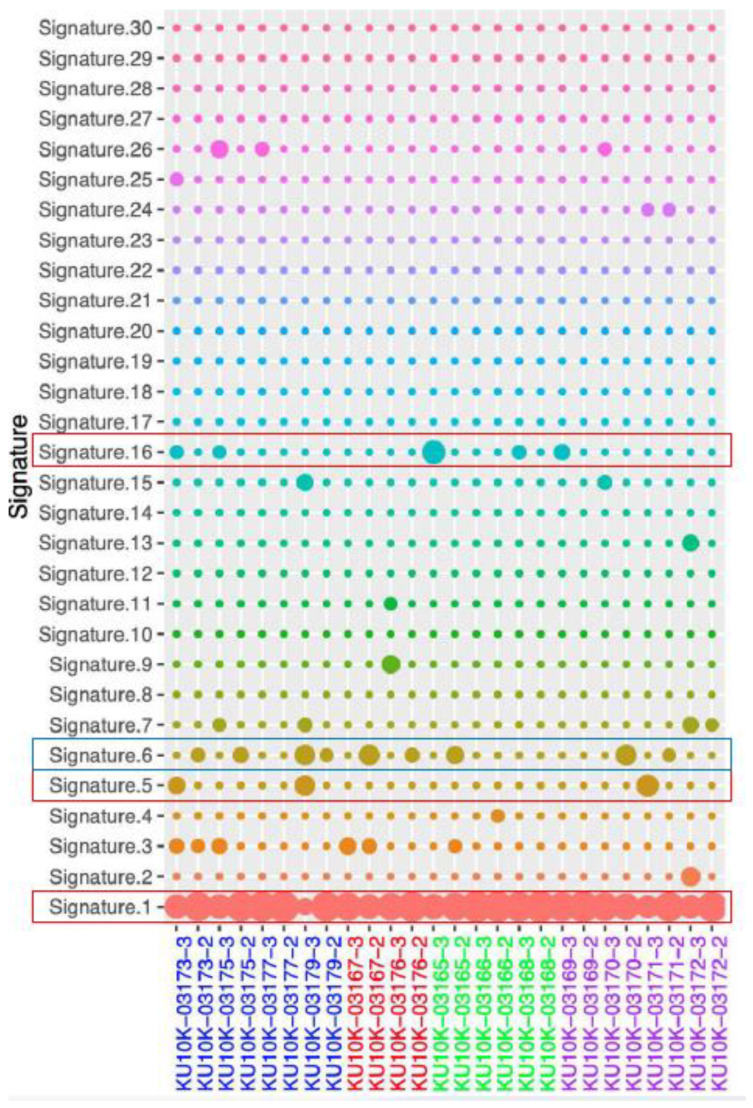
Mutational signature. Blue rectangles: dominant signal in NAT, Red rectangles: dominant signal in tumor, NAT; normal adjust to tumor.

Mutational signatures are shown in Figure 3. Age-related signature 1 was detected in both NAT and tumor tissue samples from all patients. The NAT samples from patients S170, S390, S427, S425, S429, S424, S426, and S037 harbored signature 6, which is related to defective DNA mismatch repair. In contrast, signature 5 was predominantly observed in tumor samples from patients S429, S424, and S037, whereas signature 16 was present in tumor samples from patients S423, S170, S430, S424, and S426. Although these signatures have been found in most cancer samples, their etiologies remain unknown. Ultraviolet exposure-related signature 7 was found in tumor samples from patients S426 and S037, as well as in NAT and tumor samples from patient S431. Mutation signatures 6 and 15, both associated with defective DNA mismatch repair, were found in the tumor samples from patient S037. Additionally, signatures 15 and 26 (related to DNA mismatch repair) were identified in the dCCA sample from patient S426. Moreover, signatures 2 and 13, which are related to the apolipoprotein B mRNA-editing enzyme catalytic polypeptide, were found in tumor samples from patient S431.

### 3.5. Copy Number Variation

**Figure 4:**
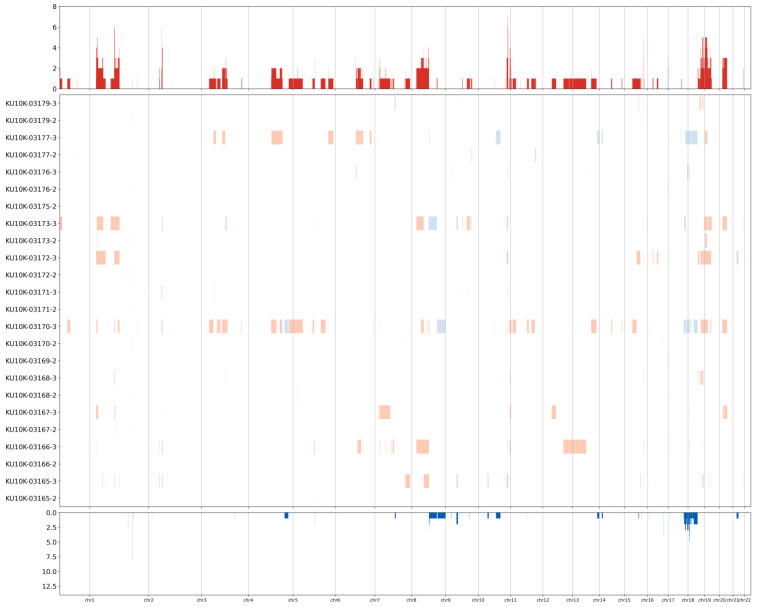
Heatmap showing the copy number variation levels. Rows represent samples and columns represent chromosomal locations. Red, amplification; blue, deletion.

CNVs of known cancer driver genes were identified in NAT and tumor tissue samples (Figure 4). Specifically, amplification of AKT serine/threonine kinase 3 (*AKT3*) and neurotrophic receptor tyrosine kinase 1 (*NTRK1*) genes on chromosome 1q was observed in tumor samples from four patients (S390, S425, S431, and S424). Amplification of *AKT2* and cyclin E1 (*CCNE1*) genes on chromosome 19q was observed in tumor tissues from four patients (S425, S431, S424, and S028). Additionally, cyclin D1 (*CCND1*; chr 11q), fibroblast growth factor receptor 3 (*FGFR3*; chr 4p), and *PIK3CA* (chr 3q) were amplified in the tumor tissues from patients S390 and S425, S425 and S028, and S425 and S424, respectively. Furthermore, *SMAD4* (chromosome 18q) was deleted in tumor tissues from patients S425 and S028. In contrast to tumor tissues, NATs did not show many gene amplifications, whereas deletions of X-chromosome genes such as lysine demethylase 5C (*KDM5C*), lysine demethylase 5C (*KDM6A*), ATRX chromatin remodeler (*ATRX*), and BCL6 corepressor (*BCOR*) were commonly observed.

## 4. Discussion

BTCs, which comprise different types of intra- and extrahepatic cholangiocarcinomas and are characterized by high mortality rates, are rare but have been increasing in incidence. In this study, genetic alterations in both NATs and tumor tissues from patients with BTCs were investigated to gain insight into the early events of disease progression and the role of the mutation profile in these processes.

The most frequently mutated genes have not been previously associated with cancer. Among these genes, *TTN*, *MUC16*, and *OBSCN*, which encode large proteins of approximately 34,000, 22,000, and 8000 amino acids, respectively, have been reported to be mutated in various types of tumors [16,17,18]. The extensive lengths of these proteins likely contribute to their high mutation rates [19]. Recent studies have linked the number of mutations in these genes to TMB, a recognized prognostic factor [16,17,18]. Although we have revealed somatic mutations in these genes shared by paired tissues from patients with BTCs, TMB was higher in tumor tissues, which is consistent with the conventional understanding of mutation accumulation during cancer development. However, the association between mutations in these genes and TMB remains unclear, owing to the limited sample size of this study.

Genomic instability plays a critical role in both the high mutation rate and acquisition of additional driver mutations that promote tumor progression [20,21]. Factors that contribute to this instability include errors in DNA replication, exposure to mutagenic agents, and defects in DNA repair mechanisms. Furthermore, the dysregulation of histone demethylases is associated with genomic instability [22]. We found that NAT samples more frequently possessed mutational signatures related to defective DNA repair than did tumor tissue samples. Frameshift mutations in the gene encoding KDM4B, a member of the lysine demethylase family, were consistently observed in the NAT samples from seven patients. Additionally, deletions in *KDM5C* and *KDM6A* were detected in the NATs. Although emerging evidence suggests that KDM enzymes play crucial roles in cancer, their effects on BTCs are not fully understood. *KDM4B* is highly expressed and functions as an oncogene in multiple types [23]. However, the roles of the *KDM4B* mutations identified in this study in BTCs have not yet been reported and warrant further investigation. In contrast, *KDM5C* and *KDM6A* have been shown to exhibit tumor-suppressive effects (e.g., metabolic repression and inhibition of cell proliferation) in intrahepatic cholangiocarcinoma [24] and hepatocellular carcinoma [16], respectively. Inactivation of *KDM5C* or *KDM6A* through deletion or mutation leads to epigenetic changes in the targeted transcription factor circuitry, disrupting tumor differentiation and promoting tumor cell proliferation [25,26]. Interestingly, we observed the loss of histone lysine demethylases KDM5C and KDM6A in NATs but not in tumor tissues, suggesting that the absence of these enzymes might be linked to the initiation of BTC development.

In addition to the identified genomic alterations, the clinicopathological findings of this study provided an important context for interpreting the mechanisms underlying biliary tract carcinogenesis. Notably, all patients with EHCCA exhibited BilIN lesions in the adjacent noncancerous bile duct epithelium. These precursor changes support the hypothesis of a multistep progression from epithelial dysplasia to invasive carcinoma. Together with the genomic data, this observation strengthens the concept of field cancerization in BTCs. The presence of BilIN in patients with EHCCA suggests that persistent inflammatory factors such as gallstones or biliary infection may create a carcinogenic microenvironment, predisposing the surrounding epithelium to acquiring somatic mutations [6,14]. Genetic profiling in this cohort revealed recurrent alterations in the DNA damage response and cell cycle regulation pathways, which are consistent with chronic epithelial stress [27,28,29].

In the tumor tissues, *TP53* was the most frequently mutated gene (6 of 13 patients). All TP53 mutations are located within the TP53 DNA-binding domain, resulting in alterations in its transcriptional function. Tumors with deficient DNA damage repair systems due to *TP53* mutations are expected to exhibit increased mutation rates and enhance cancer cell immunogenicity [30]. Additionally, *TP53* mutations co-occurring with driver gene mutations have been associated with cancer prognosis and treatment responses in previous studies [31,32,33]. In this study, each pair of *TP53* mutations in tumor tissues was observed, along with driver oncogene mutations such as those in *KRAS*, *CDKN2A*, and *SMAD4* (Supplementary Table S2). However, a direct correlation between these co-mutations and disease prognosis has not been identified. Previous studies have detected *TP53* and *KRAS* mutations in patients with advanced cholangiocarcinoma who benefited from immunotherapy, whereas patients with single *KRAS* mutations had a poorer prognosis [34].

Notably, this study revealed that patients with pathogenic mutations tended to experience recurrence or metastasis. Among them, two patients with iCCAs harboring the G12D mutation in *KRAS* developed the fastest onset of tumor metastasis following surgery, with a progression-free survival period of only 4 months for patient S428 and 7 months for patient S430. Of these two individuals, patient S428 harbored a single *KRAS* mutation and no *TP53* mutation and died 25 months later. These results are consistent with those of previous studies [34,35], indicating that the G12D mutation in *KRAS* is significantly associated with unfavorable tumor progression and can serve as a prognostic factor for BTCs. Another patient with iCCA harboring a targetable mutation (E545K in *PIK3CA*) developed liver metastases 18 months postoperatively. Three patients with pathogenic mutations in *TP53* or *CDKN2A* experienced cancer recurrence and metastasis. Therefore, patients with pathogenic mutations may have a worse prognosis, and tailored treatment options may be necessary.

Although other targetable molecular alterations, such as those in *IDH1* and *FGFR2*, were not detected in this study, cancer-related gene mutations were observed in the tumor tissues. Specifically, the PI3K–Akt signaling pathway emerged as the major pathway associated with mutated genes in tumor tissues, which is consistent with previous findings in BTCs [34]. Genes mutated in NATs were not significantly associated with cancer-related pathways. Mutations shared by NATs and tumor tissues were significantly associated with extracellular matrix–receptor interactions and focal adhesion pathways, which influence various cellular activities, such as adhesion, migration, differentiation, proliferation, and apoptosis.

Only a small fraction of interactome edges were experimentally confirmed, underscoring the speculative nature of large-scale in silico interaction datasets. Therefore, our pathway and network analyses are presented as hypothesis-generating rather than causal, emphasizing the need for future biochemical validation of candidate molecular links.

Given the limited sample size, the present findings should be interpreted as exploratory and hypothesis-generating. Validation in larger, multi-institutional cohorts will be essential to establish the generalizability of these observations. Moreover, as this study was based on static surgical specimens, it does not capture the temporal dynamics of NAT mutagenesis. Future longitudinal studies incorporating serial tissue or cfDNA analyses will be required to delineate the evolutionary trajectory from field alterations to overt malignancy. Nevertheless, these preliminary insights hold important translational potential: the identification of NAT-associated mutations could refine surgical margin assessment and inform individualized surveillance strategies. Furthermore, detecting such alterations in circulating extracellular vesicles or cfDNA may provide a non-invasive means for early detection and real-time monitoring of recurrence.

In conclusion, despite its small sample size, this study provides valuable insights into the genetic changes associated with BTC tumorigenesis. Although the role of KDMs in BTCs requires further elucidation, genomic instability in NATs appears to be an early event, with subsequently acquired cancer-related gene mutations driving the transformation to tumor status. Future studies will incorporate functional assays using BTC-derived organoids and CRISPR-based editing to determine how KDM4B, KDM5C, and KDM6A mutations contribute to epigenetic dysregulation and tumor initiation. Furthermore, targetable mutations in *KRAS* or *PIK3CA* may affect tumor recurrence and survival in patients with BTCs.

## Figures and Tables

**Table 1:** Baseline demographic and clinical characteristics of patients with biliary tract cancer.

Patient ID	Age	Sex	Diagnosis	Smoking /Alcohol	Comorbidity /Inflammatory Factors	Pathology /Differentiation	TNM stage	Margin	Associated Findings	Growth Pattern
**S424**	83	F	EHCCA	Non-smoker /None	Cholangitis	Adenocarcinoma /Moderate	pT2N0Mx	R0	BilIN-1	Diffusely infiltrating
**S426**	72	F	EHCCA	Non-smoker /None	-	Adenocarcinoma /Well	pT2N1Mx	R0	BilIN-3	Periductal infiltrating
**S028**	76	F	EHCCA	Non-smoker /None	-	Adenocarcinoma /Moderate	pT2N1Mx	R0	BilIN-3	Periductal infiltrating
**S037**	75	M	EHCCA	Ex-smoker (>1 yr) /None	-	Adenocarcinoma /Moderate	pT3N1Mx	R0	BilIN-1	Periductal infiltrating
**S427**	59	F	EHCCA	Non-smoker /None	-	Adenocarcinoma /Moderate	pT2aN1Mx	R1	IPNB with high-grade dysplasia	Periductal infiltrating
**S390**	78	M	IHCCA	Non-smoker /None	-	Adenocarcinoma /Moderate	pT3N0Mx	R1	–	Mass-forming
**S170**	57	M	IHCCA	Ex-smoker /None	Gallstone Viral hepatitis	Adenocarcinoma /Moderate	pT1aN0Mx	R0	BilIN-1	Mass-forming
**S428**	65	M	IHCCA	Smoker /Alcoholics	-	Adenocarcinoma /Moderate	pT1bN0Mx	R0	–	Mass-forming
**S430**	58	F	IHCCA	Non-smoker /None	Hepato-lithiasis	Adenocarcinoma /Moderate	pT2N0Mx	R0	–	Mixed mass-forming /periductal infiltrating
**S423**	55	F	GBC	Non-smoker /None	Gallbladder polyp	Adenocarcinoma /Moderate	pT2N0Mx	R0	–	Mixed expanding /infiltrative
**S425**	81	M	GBC	Non-smoker /None	-	Adenocarcinoma /Moderate	pT3N1Mx	R0	–	Diffuse infiltrative
**S429**	59	F	GBC	Non-smoker /Alcoholics		Adenocarcinoma /Well	pT1aN0Mx	R0		Mixed expanding /infiltrative
**S431**	77	M	GBC	Ex-smoker /None		Adenocarcinoma /Well	pT2aN1Mx	R0		Diffuse infiltrative

EHCCA, extrahepatic cholangiocarcinoma; IHCCA, intrahepatic cholangiocarcinoma; GBC, gall bladder cancer; BilIN, biliary intraepithelial neoplasia; IPNB, intraductal papillary neoplasm of the bile duct; F, female; M, male.

**Table 2:** Mutated cancer driver genes shared by NAT and tumor tissues in two or more patients.

Gene	Position	HGVS.c	HGVS.p	Variant Type	Patient ID
*APC*	chr5:112838410	c.2816A>C	p.K939T	SNP	S424
	chr5:112841706	c.6112C>G	p.L2038V	SNP	S429
*FAM131B*	chr7:143356953	c.680G>A	p.G227E	SNP	S170
	chr7:143359365	c.229G>A	p.A77T	SNP	S028
*FAM135B*	chr8:138197572	c.767G>A	p.R256H	SNP	S426
	chr8:138167985	c.1168C>T	p.P390S	SNP	S431
	chr8:138151854	c.2621A>G	p.E874G	SNP	S425
*IRS4*	chrX:108735702	c.643G>C	p.G215R	SNP	S423
	chrX:108735023	c.3322C>G	p.L1108V	SNP	S427
*MUC16*	chr19:8978644	c.2495C>T	p.S832L	SNP	S431
	chr19:8977901	c.3238A>G	p.M1080V	SNP	S427
	chr19:8906760	c.37888G>A	p.E12630K	SNP	S426
	chr19:8898625	c.39172C>T	p.P13058S	SNP	S170
	chr19:8883508	c.41501G>A	p.S13834N	SNP	S424
	chr19:8955687	c.21083T>A	p.M7028K	SNP	S428
*PREX2*	chr8:68080485	c.1685G>T	p.R562L	SNP	S429
	chr8:68108133	c.2740G>T	p.A914S	SNP	S424
*RNF213*	chr17:80346941	c.8606T>C	p.I2869T	SNP	S390
	chr17:80347169	c.8834G>A	p.R2945H	SNP	S423
*SH3GL1*	chr19:4362329	c.910C>T	p.P304S	SNP	S390
	chr19:4361726	c.981C>G	p.F327L	SNP	S170
*TCF7L2*	chr10:113141292	c.661C>A	p.P221T	SNP	S390
	chr10:113165608	c.1445C>T	p.P482L	SNP	S424
*TCF12*	chr15:57231161	c.589C>T	p.P197S	SNP	S429
	chr15:57273058	c.1774C>T	p.P592S	SNP	S037
*TP53*	chr17:7675206	c.406C>G	p.Q136E	SNP	S424
	chr17:7675161	c.451C>G	p.P151A	SNP	S427
*WNK2*	chr9:93247700	c.1700C>T	p.P567L	SNP	S429
	chr9:93289369	c.4729_4731del	p.P1577del	DEL	S430
*ZFHX3*	chr16:72959949	c.197C>T	p.A66V	SNP	S428
	chr16:72959907	c.239C>T	p.T80I	SNP	S426
	chr16:72957811	c.2335G>A	p.A779T	SNP	S428
	chr16:72788585	c.9629_9691del	p.P3210_Q3230del	DEL	S423

HGVS.c, Human Genome Variation Society coding DNA sequence; HGVS.p, Human Genome Variation Society protein sequence; Chr, chromosome.

**Table 3:** List of pathogenic or likely pathogenic mutations.

Patient ID	Gene	Position	HGVS.c	HGVS.p	ClinVar	Sample
S427	*CDKN2A*	chr9:21971209	c.151-1G>A	p.X51_splice	pathogenic	Tumor
S428	*KRAS*	chr12:25245350	c.35G>A	p.G12D	pathogenic	Tumor
S430	*KRAS*	chr12:25245350	c.35G>A	p.G12D	pathogenic	Tumor
S170	*PIK3CA*	chr3:179218303	c.1633G>A	p.E545K	pathogenic	Tumor
S427	*TP53*	chr17:7675161	c.451C>G	p.P151A	pathogenic	NAT, Tumor
S430	*TP53*	chr17:7673788	c.832C>A	p.P278T	pathogenic	Tumor
S028	*TP53*	chr17:7674191	c.772G>T	p.E258*	pathogenic	Tumor
S428	*APC*	chr5:112840210	c.4616C>A	p.S1539*	likely pathogenic	Tumor
S431	*CDKN2A*	chr9:21971037	c.322G>A	p.D108N	likely pathogenic	Tumor
S431	*ERBB2*	chr17:39711955	c.929C>T	p.S310F	likely pathogenic	Tumor
S428	*ERBB3*	chr12:56088557	c.889G>T	p.D297Y	likely pathogenic	Tumor
S170	*PTEN*	chr10:87960879	c.802-14_809del	p.X268_splice	likely pathogenic	Tumor
S028	*SMAD4*	chr18:51047214	c.170del	p.L57*	likely pathogenic	Tumor

HGVS.c, Human Genome Variation Society coding DNA sequence; HGVS.p, Human Genome Variation Society protein sequence; Chr, chromosome; NAT, normal tissue adjacent to tumor.

## Data Availability

Data pertaining to this article will be shared upon reasonable request to the corresponding authors.

## Supplementary Materials

The following supporting information can be downloaded at: https://www.mdpi.com/article/10.3390/biomedicines13112812/s1, Table S1: Pathway of mutated genes; Table S2: Outcome of patients with pathogenic mutation.

## Abbreviations

The following abbreviations are used in this manuscript:

BTCs	biliary tract cancers
BilIN	biliary intraepithelial neoplasias
BAM	binary alignment map
CGC	Cancer Gene Census
CNVs	copy number variations
dCCAs	distal cholangiocarcinomas
EHCCA	extrahepatic cholangiocarcinoma
GATK	Genome Analysis Toolkit
iCCAs	intrahepatic cholangiocarcinomas
KEGG	Kyoto Encyclopedia of Genes and Genomes
NATs	normal tissues adjacent to tumors
SNVs	single-nucleotide variants
TMB	tumor mutation burden
WES	whole-exome sequencing
WGS	whole-genome sequencing
