# Supplementary material for: Genetic Aberrations in Normal Tissues Adjacent to Biliary Tract Cancers
Source: Biomedicines. 2025 Nov 18;13(11):2812. doi: 10.3390/biomedicines13112812 (PMC12650735; doi:10.3390/biomedicines13112812)
Supplement: Supplementary file 1 [file biomedicines-13-02812-s001.zip › biomedicines-3949034-supplementary.pdf]

Supplementary Table S1. Pathway of mutated genes

	KEGG_PATHWAY_Term	-log(pvalue)	Genes
NAT	hsa01100:Metabolic pathways	1.084	KMT2E, MIOX, CYP2C8, INPP5F, GPAT3, GAPDH, NADK
Shared	hsa04512:ECM-receptor interaction	3.024	LAMA2, VWF, LAMB2, ITGB4, LAMA4, ITGA2, FN1, LAMB1, LAMC2, LAMC1, THBS4, TNN, COL6A1, SPP1, COL6A6, ITGB6, ITGA5, FREM1, FREM2
	hsa02010:ABC transporters	2.167	ABCG5, ABCA10, ABCA5, ABCA3, ABCB4, ABCB5, ABCA4, ABCA7, ABCB11, ABCA12, ABCG2
	hsa04360:Axon guidance	2.055	ROBO2, ROBO3, SEMA7A, ROCK1, NTN4, PIK3R1, EFNA5, PRKCZ, ROBO1, ABLIM3, SLIT2, PLCG1, SRGAP2, PLXNA4, PAK5, SEMA6B, SEMA4A, UNC5A, DCC, UNC5B, UNC5C, SSH2, RHOA, RGMA, ENAH, RASA1, PLXNB3, EPHA1
	hsa04510:Focal adhesion	2.002	LAMA2, ROCK1, ITGB4, LAMA4, XIAP, PDGFA, LAMC2, LAMC1, PIK3R1, ARHGAP5, ELK1, THBS4, MYLK, TNN, SPP1, PIP5K1A, FLNA, ITGB6, PAK5, VWF, LAMB2, ITGA2, FN1, LAMB1, RHOA, COL6A1, COL6A6, TLN2, ITGA5, DOCK1
	hsa05414:Dilated cardiomyopathy	1.854	MYBPC3, TGFB2, LAMA2, ITGB4, ITGA2, ADCY3, ATP2A1, CACNA1F, ADCY7, ADCY6, AGT, TTN, CACNB3, DMD, ITGB6, ITGA5, MYH7
	hsa00500:Starch and sucrose metabolism	1.827	GPI, GYS1, UGP2, HKDC1, TREH, ENPP3, PYGL, GCK, HK2
	hsa00020:Citrate cycle (TCA cycle)	1.767	ACLY, FH, MDH1, IDH2, IDH3B, SUCLG2, SDHA, PCK2
	hsa05410:Hypertrophic cardiomyopathy	1.451	MYBPC3, TGFB2, ACE, LAMA2, ITGB4, ITGA2, ATP2A1, CACNA1F, AGT, TTN, CACNB3, DMD, ITGB6, ITGA5, MYH7
	hsa04979:Cholesterol metabolism	1.381	CETP, ABCG5, NPC1, LRP1, SOAT1, PCSK9, APOA4, APOB, LPA, ABCB11
	hsa05165:Human papillomavirus infection	1.354	PATJ, LAMA2, ITGB4, LAMA4, TCF7, UBR4, LAMC2, CHD4, LAMC1, PIK3R1, PRKCZ, THBS4, WNT11, PPP2R1B, TNN, CREB3L1, HEY2, SPP1, EP300, ITGB6, HES3, IKBKE, HES4, TCF7L2, VWF, LAMB2, ITGA2, FN1, AXIN1, EIF2AK2, LAMB1, PPP2R5D, ISG15, PPP2R3A, DLG2, APC, COL6A1, COL6A6, ITGA5, TP53, ATR
Tumor	hsa05213:Endometrial cancer	6.422	MAP2K1, PTEN, BRAF, PIK3CB, AXIN2, DDB2, APC, PIK3CA, ERBB2, AKT1, CTNNB1, KRAS, POLK, RAF1, TP53
	hsa05212:Pancreatic cancer	6.376	MAP2K1, SMAD4, CDKN2A, BRAF, PIK3CB, BRCA2, TGFBR1, TGFBR2, DDB2, CDC42,

		PIK3CA, ERBB2, AKT1, KRAS, POLK, RAF1, TP53
hsa05225:Hepatocellular carcinoma	5.898	PTEN, KEAP1, PIK3CB, DVL2, AKT1, POLK, ARID2, PBRM1, MAP2K1, SMAD4, CDKN2A, IGF2, BRAF, AXIN2, ARID1A, TGFBR1, TGFBR2, DDB2, APC, PIK3CA, CTNNB1, KRAS, RAF1, TP53, CSNK1A1L
hsa05224:Breast cancer	5.797	NOTCH3, MAP2K1, NCOA3, FLT4, PTEN, BRAF, BRCA1, PIK3CB, AXIN2, BRCA2, DDB2, APC, PIK3CA, KIT, ERBB2, DVL2, AKT1, CTNNB1, KRAS, POLK, RAF1, TP53, CSNK1A1L
hsa04510:Focal adhesion	5.011	ITGB1, LAMA5, LAMA2, LAMC3, FLT4, PTEN, PIK3CB, ARHGAP5, CDC42, RELN, ERBB2, FLNA, AKT1, TNR, ITGB6, MAP2K1, ACTN1, BRAF, PARVB, COL1A1, PIK3CA, COL6A2, ITGA11, CTNNB1, COL6A6, RAF1
hsa05210:Colorectal cancer	4.942	MAP2K1, SMAD4, BRAF, PIK3CB, AXIN2, TGFBR1, TGFBR2, DDB2, APC, PIK3CA, AKT1, CTNNB1, KRAS, POLK, RAF1, TP53
hsa05220:Chronic myeloid leukemia	4.933	MAP2K1, SMAD4, CDKN1B, CDKN2A, BRAF, PIK3CB, TGFBR1, TGFBR2, DDB2, PIK3CA, AKT1, KRAS, POLK, RAF1, TP53
hsa04151:PI3K-Akt signaling pathway	4.899	ITGB1, LAMA5, IFNA4, CSF3, CDKN1B, LAMA2, LAMC3, FLT3, FLT4, CSH1, PTEN, BRCA1, PIK3CB, RELN, ERBB3, ERBB2, NTF3, AKT1, TNR, ITGB6, MCL1, MAP2K1, IGF2, COL1A1, CREB3, LPAR5, PIK3CA, COL6A2, IL2RA, ITGA11, KIT, KRAS, COL6A6, PKN1, RAF1, TP53, FGFR2
hsa05165:Human papillomavirus infection	4.745	ITGB1, LAMA5, IFNA4, NOTCH3, CDKN1B, LAMA2, LAMC3, PTEN, CHD4, PIK3CB, PTGS2, CDC42, RELN, DVL2, ATP6V0A2, AKT1, TNR, ITGB6, MAP2K1, AXIN2, COL1A1, CREB3, DLG2, APC, PIK3CA, COL6A2, ITGA11, CTNNB1, KRAS, ATM, COL6A6, MAML3, RAF1, TP53, CSNK1A1L
hsa05226:Gastric cancer	4.600	MAP2K1, SMAD4, CDKN1B, BRAF, PIK3CB, AXIN2, TGFBR1, TGFBR2, DDB2, APC, PIK3CA, ERBB2, DVL2, AKT1, CTNNB1, KRAS, POLK, RAF1, TP53, FGFR2, CSNK1A1L

Supplementary Table S2. Outcome of patients with pathogenic mutations.

Patient ID	Mutated gene	Type	Progression	PFS (months)	OS (months)
S170	*PIK3CA*	IHCCA	Liver metastasis	18	NA
S428	*KRAS*	IHCCA	Lung metastasis	4	25
S430	*KRAS, TP53*	IHCCA	Pleural metastasis	7	NA
S427	*CDKN2A, TP53*	EHCCA	Local recurrence	9	NA
S028	*TP53*	EHCCA	Liver metastasis	11	NA

IHCCA, intrahepatic cholangiocarcinoma; EHCCA, extrahepatic cholangiocarcinoma; PFS, progression-free survival; OS, overall survival; NA, not applicable.
